# How to find soluble proteins: a comprehensive analysis of alpha/beta hydrolases for recombinant expression in *E. coli*

**DOI:** 10.1186/1471-2164-6-49

**Published:** 2005-04-02

**Authors:** Markus Koschorreck, Markus Fischer, Sandra Barth, Jürgen Pleiss

**Affiliations:** 1 Institute of Technical Biochemistry, Allmandring 31, 70569 Stuttgart, Gemany

## Abstract

**Background:**

In screening of libraries derived by expression cloning, expression of active proteins in *E. coli *can be limited by formation of inclusion bodies. In these cases it would be desirable to enrich gene libraries for coding sequences with soluble gene products in *E. coli *and thus to improve the efficiency of screening. Previously Wilkinson and Harrison showed that solubility can be predicted from amino acid composition (Biotechnology 1991, 9(5):443–448). We have applied this analysis to members of the alpha/beta hydrolase fold family to predict their solubility in *E. coli*. alpha/beta hydrolases are a highly diverse family with more than 1800 proteins which have been grouped into homologous families and superfamilies.

**Results:**

The predicted solubility in *E. coli *depends on hydrolase size, phylogenetic origin of the host organism, the homologous family and the superfamily, to which the hydrolase belongs. In general small hydrolases are predicted to be more soluble than large hydrolases, and eukaryotic hydrolases are predicted to be less soluble in *E. coli *than prokaryotic ones. However, combining phylogenetic origin and size leads to more complex conclusions. Hydrolases from prokaryotic, fungal and metazoan origin are predicted to be most soluble if they are of small, medium and large size, respectively. We observed large variations of predicted solubility between hydrolases from different homologous families and from different taxa.

**Conclusion:**

A comprehensive analysis of all alpha/beta hydrolase sequences allows more efficient screenings for new soluble alpha/beta hydrolases by the use of libraries which contain more soluble gene products. Screening of hydrolases from families whose members are hard to express as soluble proteins in *E. coli *should first be done in coding sequences of organisms from phylogenetic groups with the highest average of predicted solubility for proteins of this family. The tools developed here can be used to identify attractive target genes for expression using protein sequences published in databases. This analysis also directs the design of degenerate, family- specific primers to amplify new members from homologous families or superfamilies with a high probability of soluble alpha/beta hydrolases.

## Background

It was observed that screening of libraries derived by expression cloning for new gene products with a given catalytic activity can be limited by formation of inclusion bodies. In these cases it would be desirable to construct libraries which a higher fraction of soluble gene products. Enrichment of soluble proteins could be achieved either by limiting the screening to coding sequences from phylogenetic groups with mainly soluble proteins or by the use of degenerated primers which are specific for homologous families with mainly soluble proteins.

Alternatively solubility can be improved by the use of fusion proteins [2] like NusA, MBP, Thioredoxin, GrpE, BFR, GST, DsbA [3], the N- terminal domain of IF2 [4] or phage coat protein III [5]. In addition fusion proteins allow affinity chromatography and thus simplify purification [6]. However in some cases the fusion partner has to be removed after protein purification [7] and therefore constitutes and additional step in protein preparation. Other strategies try to improve *in vivo *solubility of a recombinant protein by protein engineering using strategies like molecular evolution [8,9] or rational protein design. Examples for protein design are the insertion of positively charged residues into hydrophobic patches on the surface [10], exchange of phenylalanines by serines [11] or asparagine residues by aspartic acids [12]. It has been shown that single residues can have a major impact on solubility [13-16]. But also the expression system is important for *in vivo *solubility [2,17]. Other factors are cultivation and disruption conditions [18-20], rate of protein synthesis [21], fermentation temperature [20,22] and the amount of helper protein [12,23-26]. Fusion proteins have already been successfully used in high throughput expression- studies to improve solubility [8,27-30].

However, engineering approaches are limited to specific proteins or growth conditions and are hardly applicable to high throughput expression. Therefore, in projects where libraries are screened for activity, solubility is always an implicit criterion. Previously Harrison et al. introduced a two-parameter model based on an analysis of 81 proteins for which experimental data on solubility exists [1,3]. The model proposes that solubility of proteins in *E. coli *at physiological conditions depends mainly on protein charge and the relative number of turn-forming residues. Interestingly, the model holds for a broad variety of proteins.

We applied this model to a comprehensive analysis of the Lipase Engineering Database (LED) [31,32] which includes more than 1800 α/β-hydrolases. α/β-hydrolases share the same fold but are highly diverse in sequence. They are ubiquitous and include cellular and secreted proteins from a wide range of organisms.

The aim of this study was to find correlations between predicted solubility in *E. coli *and protein size or phylogenetic origin. Homologous families and superfamilies were analysed for the predicted solubility of their members.

## Results

### Kingdoms of life

**Figure 1:**
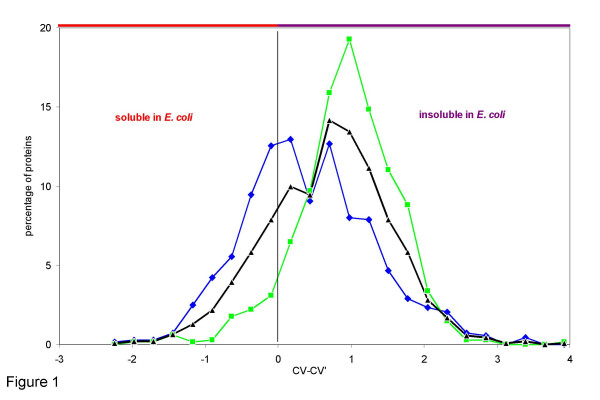
Distribution of CV-CV' of bacterial (blue), eukaryotic (green), and all (black) α/β-hydrolases.

According to Harrison et al. [1,3] the canonical variable CV-CV' predicts from the protein sequence whether a recombinantly expressed protein is soluble in the cytoplasm of *E. coli *(CV-CV' < 0) or will form inclusion bodies (CV-CV' > 0). For CV-CV' = 0 the probability of solubility is 0.5. The probability of solubility or insolubility rises for higher absolutes of CV-CV'. An analysis of the Lipase Engineering Database indicates that most of the hydrolases are predicted to be insoluble in *E. coli*, with a major peak at CV-CV' = 0.8 and a minor peak at CV-CV' = 0.2 (Figure 1). A separate analysis of hydrolases from eukaryotic and prokaryotic origin (679 and 686 hydrolases, respectively) demonstrates that these two peaks are formed predominantly by the hydrolases from each of the two kingdoms of life. The distribution of CV-CV' of bacterial hydrolases is characterized by an average of 0.42 and a first quartile of -0.18. Because the first quartile indicates the minimum solubility of the 25 % most soluble hydrolases, more than 25 % of all bacterial hydrolases are predicted to be soluble. In contrast, for eukaryotic hydrolases the average of CV-CV' is 0.94; the first quartile is 0.58. Thus, only a small fraction of eukaryotic hydrolases is predicted to be soluble in *E. coli*. α/β-hydrolases from archaea were not investigated because they are only represented with 23 hydrolases.

Thus, in general, bacterial α/β-hydrolases are predicted to be more soluble in *E. coli *than eukaryotic α/β-hydrolases.

### Protein size

**Figure 2:**
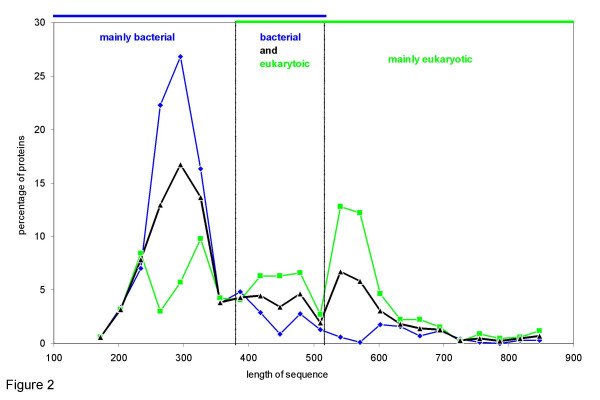
Distribution of sequence length of bacterial (blue), eukaryotic (green), and all (black) α/β-hydrolases.

**Figure 3:**
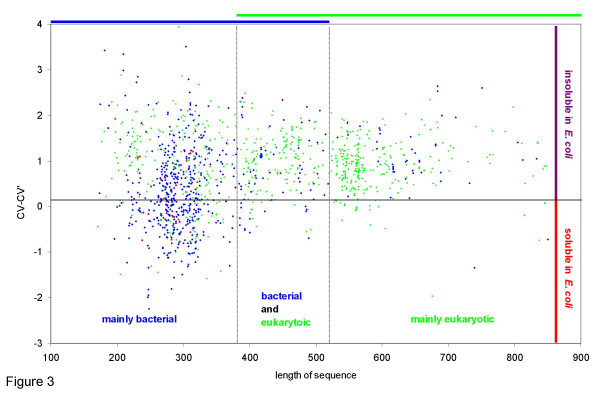
Correlation between CV-CV' and the length of protein sequences in bacterial (blue), eukaryotic (green), and archaean (red) α/β-hydrolases.

The family of α/β-hydrolases falls into three major groups of protein size: small (150–380 amino acids), medium- sized (380–520 amino acids) and large hydrolases (more than 520 amino acids) (Figure 2). Large hydrolases are mainly from eukaryotic origin while small hydrolases are mainly from bacterial origin. Correlating sequence length and predicted solubility in *E. coli *demonstrates that large hydrolases are predicted to be less soluble in *E. coli *than smaller ones (Figure 3). Hydrolases predicted to be soluble in *E. coli *(CV-CV' < 0) have sequence lengths between 200 and 400 amino acids, most hydrolases of more than 400 amino acids are predicted to be insoluble in *E. coli *(CV-CV' > 0). A high fraction of bacterial and archaean small hydrolases is predicted to be soluble in *E. coli*, while eukaryotic small hydrolases are predicted to be mainly insoluble. The two outliers with CV-CV' < -1 and a sequence length larger than 650 are a lipase from *Staphylococcus xylosus *(AAG35726) and CG6296 from *Drosophila melanogaster *(AAF56648). Both are putative proteins and have not yet been expressed in *E. coli*. Thus, there are two general observations: (1) small α/β-hydrolases are predicted to be more soluble in *E. coli *than large α/β-hydrolases and (2) eukaryotic α/β-hydrolases are larger than prokaryotic α/β-hydrolases.

### Analysis of groups formed by protein size

To distinguish the effects of hydrolase size and phylogenetic origin on solubility, small, medium- sized and large α/β-hydrolases were investigated separately, and hydrolases were grouped by phylogeny of their origin (Tables 1, 2, 3).

**Table 1:** Hydrolases from 171 to 379 amino acids sorted by taxa of their host n is the number of hydrolases in the group, charge the charge per residue, turn the relative number of turn- forming residues. Only groups with at least eight hydrolases were included.

**Taxon**	**n**	**CV-CV' average**	**CV-CV' first quartile**	**Charge average**	**Turn average**	**Length average**
d-proteobacteria	8	0.02	-0.87	-0.062	0.230	267
enterobacteria	29	0.02	-0.28	-0.061	0.229	287
a-proteobacteria	66	0.14	-0.26	-0.054	0.223	300
actinobacteria	127	0.23	-0.49	-0.060	0.240	291
proteobacteria	302	0.28	-0.23	-0.052	0.230	293
eubacteria	558	0.29	-0.28	-0.050	0.229	288
archaea	22	0.30	-0.43	-0.052	0.229	272
g-proteobacteria	149	0.31	-0.27	-0.052	0.232	291
b-proteobacteria	46	0.55	-0.04	-0.044	0.236	298
plants	52	0.57	-0.23	-0.048	0.239	301
ascomycetes	40	0.89	0.32	-0.046	0.257	267
fungi	43	0.91	0.37	-0.046	0.258	268
eukaryota	235	0.94	0.36	-0.040	0.250	284
chordata	72	0.98	0.85	-0.036	0.247	269
mammalia	65	1.00	0.88	-0.035	0.245	267
metazoa	140	1.10	0.78	-0.035	0.252	283
arthropoda	48	1.38	0.86	-0.031	0.263	300

**Table 2:** Hydrolases from 380 to 519 amino acids sorted by taxa of their host n is the number of hydrolases in the group, charge the charge per residue, turn the relative number of turn- forming residues. Only groups with at least eight hydrolases were included.

**Taxon**	**n**	**CV-CV' average**	**CV-CV' first quartile**	**Charge average**	**Turn average**	**Length average**
actinobacteria	15	0.30	-0.33	-0.060	0.246	439
ascomycetes	15	0.52	0.09	-0.047	0.235	447
fungi	21	0.67	0.25	-0.046	0.242	435
eubacteria	79	0.84	0.30	-0.044	0.251	437
proteobacteria	38	0.89	0.44	-0.042	0.250	436
g-proteobacteria	11	0.96	0.59	-0.055	0.279	454
arthropoda	40	1.04	0.50	-0.034	0.246	441
eukaryota	165	1.04	0.72	-0.035	0.246	443
a-proteobacteria	19	1.08	0.56	-0.030	0.239	439
metazoa	136	1.08	0.76	-0.033	0.245	444
chordata	76	1.25	0.99	-0.030	0.249	448
mammalia	71	1.25	0.98	-0.031	0.251	446
plants	8	1.42	1.10	-0.034	0.268	452

**Table 3:** Hydrolases larger than 519 amino acids sorted by taxa of their host n is the number of hydrolases in the group, charge the charge per residue, turn the relative number of turn- forming residues. Only groups with at least eight hydrolases were included.

**Taxon**	**n**	**CV-CV' average**	**CV-CV' first quartile**	**Charge average**	**Turn average**	**Length average**
arthropoda	98	0.75	0.51	-0.048	0.251	606
metazoa	262	0.85	0.56	-0.046	0.254	649
eukaryota	279	0.88	0.58	-0.045	0.255	644
mammalia	95	0.88	0.56	-0.046	0.256	720
chordata	108	0.90	0.58	-0.046	0.257	705
enterobacteria	8	0.99	0.74	-0.054	0.279	633
eubacteria	49	1.12	0.76	-0.047	0.276	654
g-proteobacteria	32	1.17	0.79	-0.049	0.285	643
ascomycetes	13	1.21	0.88	-0.046	0.278	552
proteobacteria	35	1.22	0.79	-0.048	0.286	638
fungi	14	1.25	0.88	-0.045	0.279	553

Small hydrolases have an average of CV-CV' of 0.48 and a first quartile of -0.17, thus more than 25 % of small hydrolases are predicted to be soluble in *E. coli*. All taxa have a positive average of CV-CV' (Table 1), indicating that less than 50 % of the hydrolases in each taxon are predicted to be soluble. Hydrolases from eukaryotic taxa are at average predicted to be highly insoluble. All prokaryotic taxa and plants have negative first quartiles of CV-CV', thus more than 25 % of their hydrolases are predicted to be soluble. All other eukaryotic taxa have positive first quartiles of CV-CV', thus most of their hydrolases are predicted to be insoluble. The taxa containing hydrolases with the highest predicted solubility average are from bacteria, the taxa containing hydrolases with the lowest predicted solubility average are from eukaryota.

Medium- sized hydrolases have an average of CV-CV' of 0.98 and a first quartile of 0.54. Hydrolases of all taxa have a positive average of CV-CV', and thus are mainly predicted to be insoluble (Table 2). Only actinobacteria have a negative first quartile of CV-CV', indicating that more than 25 % of its hydrolases are predicted to be soluble, all other taxa have positive first quartiles of CV-CV'. The taxa containing hydrolases with the highest predicted solubility average are from bacteria and fungi. The taxa containing hydrolases with the lowest predicted solubility average are from metazoa and plants.

Large hydrolases have an average of CV-CV' of 0.93 and a first quartile of 0.61, they are in general predicted to be highly insoluble. All taxa have positive averages and first quartiles of CV-CV', and thus most of their hydrolases are predicted to be insoluble (Table 3). The taxa containing hydrolases with the highest predicted solubility average are from metazoa, the taxa containing hydrolases with the lowest predicted solubility average are from bacteria and fungi.

In general CV-CV' from bacterial hydrolases is much lower if the hydrolase is small, while metazoan hydrolases have a lower average and first quartile of CV-CV' if the hydrolase is large. Though large metazoan hydrolases have a higher probability of solubility than small metazoan hydrolases, few large hydrolases are predicted to be soluble in *E. coli *(Figure 3). This is consistent with the result from Table 3 that in the analysis of large hydrolases no taxon with a negative first quartile of CV-CV' could be found.

Thus there are several conclusions. Large α/β-hydrolases from bacteria are predicted to be less soluble than smaller ones. Small bacterial α/β-hydrolases are predicted to be more soluble than both small and large eukaryotic α/β-hydrolases. But large hydrolases from metazoa are predicted to be more soluble than large hydrolases from bacteria and small hydrolases from metazoa. So there seems to be a principal difference between metazoa and bacteria. Fungi, especially ascomycetes, behave differently. Their α/β-hydrolases with the highest predicted solubility are mainly medium- sized.

### Analysis of solubility by genera

Of the 257 genera represented in the database most include only a few hydrolases. Therefore the 29 genera with at least ten hydrolases were analyzed (Table A in the supplementary file 1). Most hydrolases of all eukaryotic taxa with the exception of *Oryza *are predicted to be highly insoluble in *E. coli*.

Hydrolases from bacterial genera show a wide range of averages of predicted solubility. Interestingly the bacterial genera with the highest and the lowest average of CV-CV' (*Mycobacterium *and *Rhodococcus*) are both from actinobacteria. The third large genus of actinobacteria, *Streptomyces*, is predicted to include more than 25 % soluble hydrolases as it is shown by a negative first quartile.

Thus the averages of different genera of a taxon may differ completely in predicted solubility. Therefore a strategy to identify α/β-hydrolases from actinobacteria that are soluble in *E. coli *should focus on proteins from *Rhodococcus *and *Streptomyces*, but not from *Mycobacterium*.

### Analysis of solubility by sequence similarity

Hydrolases with sequence similarity have been assigned to superfamilies which were analysed (Table B in the supplementary file 1). Superfamilies with mostly hydrolases of high predicted solubility contain mainly intracellular α/β-hydrolases from bacteria (supplementary file 2). The averages of CV-CV' of the superfamilies that contain almost exclusively bacterial hydrolases range from -0.77 to 0.36. Metazoan, fungal and secreted hydrolases can mainly be found in superfamilies with averages of CV-CV' between 0.38 (hormone sensitive lipases) and 1.45 (cutinases) (supplementary file 2). In general, the average of CV-CV' depends on sequence length. However, the superfamily with the lowest sequence length in this table (Table B in the supplementary file 1), *Bacillus *lipase, has the lowest predicted solubility.

Proteins from cytosolic hydrolases, a large superfamily that contains mainly epoxide hydrolases and haloalkane dehalogenases, are in general predicted to be more soluble than proteins from most other superfamilies. The average of CV-CV' is 0.29 and the first quartile is -0.22. This superfamily was chosen for a more detailed analysis of homologous families (Table C in the supplementary file 1). The CV-CV' averaged over hydrolases of each homologous family of this superfamily ranges from -0.29 to 0.86. This shows that there can be the prediction of families with mostly soluble and of families with mostly insoluble proteins in one superfamily. This observation is analogous to the previous observation with genera and higher taxa. As expected the homologous families with the highest predicted solubility average are dominated by bacterial hydrolases and the families with proteins of low predicted solubility rather contain eukaryotic hydrolases (supplementary file 2). The homologous family with the highest predicted solubility average, which is dominated by eukaryots, is the 'soluble plant epoxide hydrolases'. Almost 50 % of its members are predicted to be soluble in *E. coli*.

Thus the averages of different homologous families of a superfamily may differ significantly in predicted solubility.

## Discussion

### The solubility model

The dataset of the statistical solubility model were 81 highly diverse proteins, for which solubility data exists, from a wide range of organisms [1]. The published prediction accuracy of the five parameter model was 76 % for soluble proteins and 91 % for insoluble proteins [1], the overall accuracy for solubility prediction of proteins from the dataset was 88% [1]. It has been discovered that only two out of the five parameters are critical for distinguishing between soluble and insoluble proteins [3]. Therefore, CV-CV', the indicator of predicted solubility, was derived by two terms: the total charge calculated by the relative numbers of arginines, lysines, aspartic acids and glutamic acids, and the relative number of turn forming residues calculated by the relative number of asparagines, glycines, prolines and serines. Those two parameters show a level of significance of 100% [1].

Most of the variation of CV-CV' among α/β-hydrolases is caused by the charge term. A high negative or positive charge results in the best predicted solubility. However, the pK value of a titratable group is highly dependent on the proteins structure. Short- and long-range interactions can lead to pK shifts of more than two units, changing the total protein charge by more than five unit charges [33,34].

An evaluation of nine frequently used fusion proteins used to improve solubility in *E. coli *[3-5] showed that seven proteins indeed are predicted to be soluble in *E. coli *(data not shown). The exceptions are MBP which has a CV-CV' of 0.23, and phage coat protein III which has a CV-CV' of 0.62 (data not shown).

To test the model in the prediction of solubility of hydrolases, 35 hydrolases from the PDB which were annotated as expressed in *E. coli *were examined (Table D in the supplementary file 1). Because good solubility is prerequisite for successful crystallization, this group of proteins served as a positive control for the predictive value of our method. 24 of 35 hydrolases are predicted to be soluble in *E. coli *(CV-CV' < 0). For all 35 hydrolases, the average of CV-CV' is -0.23, the first quartile is -0.80. Thus, the predicted average solubility of this group of proteins is much higher than any single protein family in the database, including proteins from enterobacteria and the genus *Escherichia *which have an average of CV-CV' of 0.24 and 0.10 respectively (data not shown), which is a strong support for the reliability of this method.

In addition, the observation that substitution of asparagines by aspartic acids in DsbA-IGFBP-3 fusion proteins improved solubility [12] is consistent with the solubility formula because the fusion protein is already predicted to be negatively charged (data not shown). Thus, increasing the negative charge increases predicted solubility. Similarly, incorporation of solvent exposed positive charged amino acids improved solubility of consensus ankyrin repeat proteins [10]. Here the net charge proposed by the solubility formula was zero (data not shown), so solubility is predicted to be increased by insertion of positively or negatively charged amino acids.

However, the solubility formula exclusively depends on sequence information and neglects the structural context. Therefore, in some cases this simple model resulted in wrong predictions. In the model, substitution of multiple phenylalanine residues by serine [11] led to a predicted increase of the ratio of turn-forming residues in the formula and thus to a lowered predicted solubility in *E. coli*. Instead, an increase in solubility was observed experimentally [11] which can be explained by the structural context: replacement of solvent- accessible phenylalanines increased polarity of the proteins surface and thus its solubility. The fact that single residues can have a huge impact on *in vivo *solubility [13-16] is not always fully explainable by the solubility formula. Therefore, for the design of proteins with higher solubility the formula should be combined with a careful investigation of the structural context.

### Solubility of α/β-hydrolases

Protein size was not included as a parameter in the solubility model, because it showed no significant difference between soluble and insoluble proteins of the dataset [1]. However, the analysis of groups formed by α/β-hydrolase size revealed that both protein size and phylogeny of organisms have a major impact on predicted solubility. Statements like 'Small hydrolases are predicted to be more soluble than large hydrolases', 'Bacterial hydrolases are predicted to be more soluble than eukaryotic hydrolases' are generally true, but simplifications.

For small to medium- sized hydrolases, bacterial hydrolases are predicted to be more soluble than eukaryotic hydrolases, archaea are somewhere in between. The relatively high predicted solubility of medium- sized fungal hydrolases is in contrast to the low predicted solubility of small and large fungal hydrolases. Hydrolases from plants are predicted to be much more soluble when they are small than when they are of medium size. However, the significance of this statement is relatively low because there are only eight medium- sized hydrolases from plants.

Though large hydrolases are mainly predicted to be insoluble the CV-CV' values are much lower for metazoan than for bacterial and fungal hydrolases. So here the situation is opposite to the situation of small and medium- sized hydrolases. Additionally, large metazoan hydrolases are predicted to be more soluble in *E. coli *than small and medium- sized metazoan hydrolases.

These results propose that, in problematic cases where soluble hydrolases are very rare, it makes sense to screen for large hydrolases in coding sequences of metazoa, small hydrolases should be searched in bacterial or archaean and medium- sized hydrolases in actinobacterial or ascomycetic coding sequences. Secreted and eukaryotic hydrolases generally have a low predicted solubility in *E. coli*.

As the data about taxa, the family information could be used to efficiently search for new soluble hydrolases. If a specific catalytic activity is observed in different homologous families or a soluble member of a specific superfamily is searched for a structural genomics project, family specific degenerate primers could preferably be designed for families which are predicted to include mainly soluble hydrolases.

If soluble hydrolases of a given size are rarely found, screening of libraries could be limited to coding sequences from taxa where soluble hydrolases are expected.

## Conclusion

General rules for the relationship between predicted solubility in *E. coli*, protein size and phylogenetic origin are:

1) Bacterial hydrolases are predicted to be more soluble in *E. coli *than eukaryotic hydrolases.

2) Small hydrolases are predicted to be more soluble in *E. coli *than large hydrolases.

3) In one taxon huge differences of predicted solubility between genera can exist.

4) In one superfamily huge differences of predicted solubility between homologous families can exist.

When taking into account the groups formed by protein size there are three additional rules:

5) Small bacterial hydrolases are predicted to be more soluble than small and large hydrolases from eukaryotes and large bacterial hydrolases.

6) Large metazoan hydrolases are predicted to be more soluble than large hydrolases from bacteria and small hydrolases from metazoa.

7) Fungal medium- sized hydrolases are predicted to be more soluble than small or large hydrolases from fungi.

When using the family characteristics family- specific primers could be designed to amplify members from specific homologous families with high averages of predicted solubility in *E. coli*.

## Methods

### The database

Sequence data for analysis was derived from the Lipase Engineering Database (LED) [31,32] which integrates sequence, structure and annotation information of α/β-hydrolases. The database comprises 1820 hydrolases which are grouped into 149 homologous families and 45 superfamilies.

To remove fragments, only hydrolases larger than 170 amino acids were included in the analysis.

### The solubility model

A two parameter statistical model by Harrison et al. [1,3] was used to predict *in vivo *solubility of recombinant proteins in *E. coli*. The main parameters for solubility in *E. coli *are the relative number of turn forming residues (asparagine, glycine, proline and serine) and the absolute of charge per residue which is determined by the fraction of positively and negatively charged amino acids (arginine, lysine, aspartic acid, glutamic acid). These values have been combined to a canonical variable CV, where N, G, P, S, R, K, D, E, are the numbers of asparagines, glycines, prolines, serines, arginines, lysines, aspartic acids and glutamic acids, respectively, and n is the total number of residues in the sequence.



To distinguish soluble from insoluble protein a threshold of CV' = 1.71 was introduced. If the difference CV-CV' is smaller than zero, the protein is predicted to be soluble in *E. coli*. If it is larger than zero the protein is predicted to be insoluble in *E. coli*. From CV-CV' a probability of solubility is calculated:

*P *= 0.4934 + 0.276|*CV - CV*'| - 0.0392(*CV - CV*')^2^

The higher the absolute of CV-CV', the higher the probability of solubility (CV-CV' < 0) or insolubility (CV-CV' > 0). CV-CV' values of -0.4, 0.0 and 1.1 indicate probabilities of solubility of 60 %, 50 % and 25 %, respectively.

The dependency of solubility on the parameters relative number of turn forming residues and absolute of charge per residue can be interpreted as follows: the higher the charge, the higher the repulsion between proteins, thus preventing aggregation. Additionally, many turn-forming residues slow down protein folding, resulting in a high concentration of folding intermediates which can form aggregates.

### Statistical methods

Averages of CV-CV', the relative number of turn forming residues, charge per residue and length of protein sequence were computed. Additionally first quartiles of CV-CV' were determined. For determination of first quartiles the values were ordered by size and divided into four groups with the same number of members. The first quartile is the largest value of CV-CV' of the group which contains 25 % of the smallest values of CV-CV'. This means that 25 % of all proteins in the distribution are predicted to have at least the solubility that is represented by the value of the first quartile. When used in combination with averages, quartiles give additional information about the shape of the distribution. They make it possible to compare distributions even if their averages are very similar. While the average value of CV-CV' gives an upper limit of solubility to 50 % of all proteins of a distribution, the first quartile indicates the solubility of the 25 % best soluble proteins.

### Visualisation of distributions

The optimal window size for the visualisation of the distributions d_1_, d_2 _and d_3 _(Figures 1 and 2) was determined as follows. For each distribution a window size w was determined, where max and min are the largest and the smallest values of the distribution and n is the number of values in the distribution.



As an overall window size for the graphics the largest of the three window sizes was taken.

## Abbreviations

*E. coli*, *Escherichia coli*.

## Authors' contributions

MK wrote the Perl scripts, carried out the analysis and drafted the manuscript. MF provided the database; SB participated in the design of the study. JP supervised the study. All authors read and approved the final manuscript.

## Supplementary Material

Additional File 1 All tables which are too large to be included in the main article. The format is Microsoft Word 2000. Click here for file

Additional File 2 All hydrolases in the analysis were grouped by homologous families and superfamilies. The format is UNIX plain text. Click here for file

## References

[B1] Flaschel E, Friehs K (1993). Improvement of downstream processing of recombinant proteins by means of genetic engineering methods. Biotechnol Adv.

[B2] Davis GD, Elisee C, Newham DM, Harrison RG (1999). New fusion protein systems designed to give soluble expression in Escherichia coli. Biotechnol Bioeng.

[B3] Sorensen HP, Sperling-Petersen HU, Mortensen KK (2003). A favorable solubility partner for the recombinant expression of streptavidin. Protein Expr Purif.

[B4] Jensen KB, Larsen M, Pedersen JS, Christensen PA, Alvarez-Vallina L, Goletz S, Clark BF, Kristensen P (2002). Functional improvement of antibody fragments using a novel phage coat protein III fusion system. Biochem Biophys Res Commun.

[B5] Terpe K (2003). Overview of tag protein fusions: from molecular and biochemical fundamentals to commercial systems. Appl Microbiol Biotechnol.

[B6] Jenny RJ, Mann KG, Lundblad RL (2003). A critical review of the methods for cleavage of fusion proteins with thrombin and factor Xa. Protein Expr Purif.

[B7] Waldo GS (2003). Genetic screens and directed evolution for protein solubility. Curr Opin Chem Biol.

[B8] Ito Y, Kawama T, Urabe I, Yomo T (2004). Evolution of an arbitrary sequence in solubility. J Mol Evol.

[B9] Mosavi LK, Peng ZY (2003). Structure-based substitutions for increased solubility of a designed protein. Protein Eng.

[B10] Murby M, Samuelsson E, Nguyen TN, Mignard L, Power U, Binz H, Uhlen M, Stahl S (1995). Hydrophobicity engineering to increase solubility and stability of a recombinant protein from respiratory syncytial virus. Eur J Biochem.

[B11] Zhang Y, Olsen DR, Nguyen KB, Olson PS, Rhodes ET, Mascarenhas D (1998). Expression of eukaryotic proteins in soluble form in Escherichia coli. Protein Expr Purif.

[B12] Izard J, Parker MW, Chartier M, Duche D, Baty D (1994). A single amino acid substitution can restore the solubility of aggregated colicin A mutants in Escherichia coli. Protein Eng.

[B13] Wetzel R (1994). Mutations and off-pathway aggregation of proteins. Trends Biotechnol.

[B14] Wetzel R, Perry LJ, Veilleux C (1991). Mutations in human interferon gamma affecting inclusion body formation identified by a general immunochemical screen. Biotechnology (N Y).

[B15] Dale GE, Broger C, Langen H, D'Arcy A, Stuber D (1994). Improving protein solubility through rationally designed amino acid replacements: solubilization of the trimethoprim-resistant type S1 dihydrofolate reductase. Protein Eng.

[B16] Makrides SC (1996). Strategies for achieving high-level expression of genes in Escherichia coli. Microbiol Rev.

[B17] Gonzalez C, Lagos R, Monasterio O (1996). Recovery of soluble protein after expression in Escherichia coli depends on cellular disruption conditions. Microbios.

[B18] Hart RA, Lester PM, Reifsnyder DH, Ogez JR, Builder SE (1994). Large scale, in situ isolation of periplasmic IGF-I from E. coli. Biotechnology (N Y).

[B19] MacDonald LM, Armson A, Thompson RC, Reynoldson JA (2001). Expression of Giardia duodenalis beta-tubulin as a soluble protein in Escherichia coli. Protein Expr Purif.

[B20] Kiefhaber T, Rudolph R, Kohler HH, Buchner J (1991). Protein aggregation in vitro and in vivo: a quantitative model of the kinetic competition between folding and aggregation. Biotechnology (N Y).

[B21] Ingley E, Hemmings BA (1999). Large-scale expression and purification of a soluble form of the pleckstrin homology domain of the human protooncogenic serine/threonine protein kinase PKB (c-akt) in Escherichia coli. Protein Expr Purif.

[B22] Buchner J, Rudolph R (1991). Routes to active proteins from transformed microorganisms. Curr Opin Biotechnol.

[B23] Yokoyama K, Kikuchi Y, Yasueda H (1998). Overproduction of DnaJ in Escherichia coli improves in vivo solubility of the recombinant fish-derived transglutaminase. Biosci Biotechnol Biochem.

[B24] Caspers P, Stieger M, Burn P (1994). Overproduction of bacterial chaperones improves the solubility of recombinant protein tyrosine kinases in Escherichia coli. Cell Mol Biol (Noisy-le-grand).

[B25] Thomas JG, Ayling A, Baneyx F (1997). Molecular chaperones, folding catalysts, and the recovery of active recombinant proteins from E. coli. To fold or to refold. Appl Biochem Biotechnol.

[B26] Hammarstrom M, Hellgren N, van Den Berg S, Berglund H, Hard T (2002). Rapid screening for improved solubility of small human proteins produced as fusion proteins in Escherichia coli. Protein Sci.

[B27] Kawasaki M, Inagaki F (2001). Random PCR-based screening for soluble domains using green fluorescent protein. Biochem Biophys Res Commun.

[B28] Nakayama M, Ohara O (2003). A system using convertible vectors for screening soluble recombinant proteins produced in Escherichia coli from randomly fragmented cDNAs. Biochem Biophys Res Commun.

[B29] Goda N, Tenno T, Takasu H, Hiroaki H, Shirakawa M (2004). The PRESAT-vector: asymmetric T-vector for high-throughput screening of soluble protein domains for structural proteomics. Protein Sci.

[B30] Wilkinson DL, Harrison RG (1991). Predicting the solubility of recombinant proteins in Escherichia coli. Biotechnology (N Y).

[B31] Fischer M, Pleiss J (2003). The Lipase Engineering Database: a navigation and analysis tool for protein families. Nucleic Acids Res.

[B32] Barth S, Fischer M, Schmid RD, Pleiss J (2004). The database of epoxide hydrolases and haloalkane dehalogenases: one structure, many functions. Bioinformatics.

[B33] Alexov EG, Gunner MR (1997). Incorporating protein conformational flexibility into the calculation of pH-dependent protein properties. Biophys J.

[B34] Alexov EG, Gunner MR (1999). Calculated protein and proton motions coupled to electron transfer: electron transfer from QA- to QB in bacterial photosynthetic reaction centers. Biochemistry.

