# Supplementary material for: How to find soluble proteins: a comprehensive analysis of alpha/beta hydrolases for recombinant expression in E. coli
Source: BMC Genomics. 2005 Apr 2;6:49. doi: 10.1186/1471-2164-6-49 (PMC1079826; doi:10.1186/1471-2164-6-49)
Supplement: Additional File 1 — All tables which are too large to be included in the main article. The format is Microsoft Word 2000. [file 1471-2164-6-49-S1.doc]

## Table A - Proteins larger than 170 amino acids sorted by the genus of their host

Only genera with at least 10 proteins were included, otherwise the number of genera would have been 257. n is the number of proteins in the group, charge the charge per residue, turn the relative number of turn- forming residues. Plants are highlighted in green, metazoa in red and fungi in blue. Bacteria are not highlighted.

| **taxon** | **n** | **CV-CV’**  **average** | CV-CV' **first quartile** | **charge**  **average** | **turn**  **average** | **length**  **average** |
| --- | --- | --- | --- | --- | --- | --- |
| *Rhodococcus* | 26 | -0.22 | -0.70 | -0.074 | 0.239 | 313 |
| *Sphingomonas* | 11 | -0.05 | -0.61 | -0.062 | 0.226 | 271 |
| *Rhizobium* | 13 | 0.02 | -0.27 | -0.058 | 0.222 | 287 |
| *Photorhabdus* | 10 | 0.09 | -0.38 | -0.064 | 0.240 | 375 |
| *Escherichia* | 11 | 0.10 | -0.56 | -0.063 | 0.238 | 267 |
| *Nostoc* | 11 | 0.17 | -0.41 | -0.049 | 0.216 | 289 |
| *Streptomyces* | 59 | 0.20 | -0.49 | -0.059 | 0.237 | 315 |
| *Ralstonia* | 19 | 0.21 | -0.47 | -0.051 | 0.223 | 318 |
| *Synechocystis* | 10 | 0.27 | 0.03 | -0.055 | 0.234 | 286 |
| *Oryza* | 15 | 0.31 | -0.67 | -0.054 | 0.234 | 300 |
| *Staphylococcus* | 16 | 0.38 | -0.46 | -0.056 | 0.242 | 543 |
| *Geobacillus* | 10 | 0.38 | -1.10 | -0.050 | 0.231 | 346 |
| *Xylella* | 14 | 0.39 | -0.24 | -0.048 | 0.228 | 335 |
| *Burkholderia* | 19 | 0.41 | -0.02 | -0.044 | 0.233 | 310 |
| *Salmonella* | 11 | 0.45 | -0.12 | -0.049 | 0.234 | 404 |
| *Acinetobacter* | 10 | 0.48 | -0.28 | -0.049 | 0.235 | 316 |
| *Pseudomonas* | 110 | 0.52 | -0.13 | -0.052 | 0.247 | 341 |
| *Caulobacter* | 20 | 0.68 | -0.48 | -0.039 | 0.230 | 406 |
| *Bacillus* | 32 | 0.75 | -0.32 | -0.040 | 0.246 | 319 |
| *Mycoplasma* | 25 | 0.81 | 0.64 | -0.013 | 0.215 | 277 |
| *Mycobacterium* | 42 | 0.82 | -0.01 | -0.043 | 0.247 | 311 |
| *Caenorhabditis* | 87 | 0.91 | 0.42 | -0.039 | 0.246 | 521 |
| *Mus* | 86 | 0.93 | 0.72 | -0.038 | 0.245 | 461 |
| *Drosophila* | 127 | 0.95 | 0.63 | -0.041 | 0.252 | 483 |
| *Rattus* | 34 | 0.96 | 0.80 | -0.042 | 0.253 | 562 |
| *Oryctolagus* | 12 | 0.99 | 0.78 | -0.038 | 0.249 | 462 |
| *Arabidopsis* | 36 | 1.09 | 0.61 | -0.038 | 0.254 | 346 |
| *Schizosaccharomyces* | 10 | 1.10 | 0.71 | -0.030 | 0.240 | 370 |
| *Homo* | 56 | 1.12 | 0.75 | -0.036 | 0.252 | 470 |

## Table B - All proteins of the database grouped by superfamilies

n is the number of proteins in the group, charge the charge per residue, turn the relative number of turn- forming residues, sID the superfamily identifier (see Table C). The cytosolic hydrolases superfamily is identified by red colouring. The description in parentheses indicates the types of protein in the respective superfamily. Only superfamilies with at least four proteins were included.

| **sID** | **superfamily** | **n** | **CV-CV’**  **average** | **CV-CV' first quartile** | **charge**  **average** | **turn**  **average** | **length**  **average** |
| --- | --- | --- | --- | --- | --- | --- | --- |
| 30 | Sfam based on GI 729450  (carboxylesterases) | 17 | -0.77 | -1.58 | -0.074 | 0.204 | 252 |
| 23 | Sfam based on GI 126520  (acyltransferases) | 16 | -0.24 | -0.38 | -0.069 | 0.228 | 307 |
| 36 | Sfam based on GI 38490076  (enol-lactone hydrolases and acyltransferases) | 19 | -0.14 | -0.54 | -0.060 | 0.216 | 299 |
| 22 | Sfam based on GI 2829433  (proline iminopeptidases) | 48 | -0.07 | -0.46 | -0.057 | 0.218 | 315 |
| **20** | **Cytosolic Hydrolases** | **302** | **0.29** | **-0.22** | **-0.050** | **0.226** | **312** |
| 32 | Sfam based on GI 22957072  (esterases) | 4 | 0.36 | 0.09 | -0.065 | 0.259 | 226 |
| 16 | Hormone sensitive lipases | 67 | 0.38 | -0.32 | -0.049 | 0.230 | 420 |
| 1 | Filamentous fungi lipases | 35 | 0.45 | 0.05 | -0.053 | 0.242 | 317 |
| 35 | Sfam based on GI 7470860  (serine esterases) | 5 | 0.46 | 0.03 | -0.064 | 0.263 | 207 |
| 25 | Sfam based on GI 21647874  (esterases) | 4 | 0.48 | -0.13 | -0.035 | 0.228 | 235 |
| 3 | Non-heme peroxidases | 100 | 0.52 | 0.03 | -0.037 | 0.223 | 287 |
| 12 | *Acinetobacter* esterases | 26 | 0.62 | -0.12 | -0.039 | 0.227 | 317 |
| 27 | Sfam based on GI 729942  (lipases and others) | 25 | 0.80 | 0.67 | -0.056 | 0.270 | 522 |
| 28 | Sfam based on GI 1430921  (lipases, partly secreted) | 7 | 0.82 | 0.10 | -0.044 | 0.248 | 347 |
| 9 | Gastric lipases | 68 | 0.85 | 0.33 | -0.036 | 0.238 | 425 |
| 19 | Microsomal Hydrolases | 30 | 0.86 | 0.47 | -0.039 | 0.241 | 426 |
| 2 | Carboxylesterases | 310 | 0.88 | 0.52 | -0.045 | 0.255 | 584 |
| 24 | Sfam based on GI 3023719  (carboxylesterases and lysophospholipases) | 80 | 0.97 | 0.32 | -0.044 | 0.257 | 244 |
| 34 | Sfam based on GI 7520955  (esterase) | 4 | 0.97 | 0.74 | -0.003 | 0.179 | 273 |
| 29 | Sfam based on GI 11992014  (esterases, lipases and peptide hydrolases) | 41 | 1.00 | 0.57 | -0.038 | 0.247 | 413 |
| 7 | *Pseudomonas* lipases | 14 | 1.02 | 0.68 | -0.069 | 0.310 | 539 |
| 5 | *Burkholderia* lipases | 41 | 1.27 | 1.01 | -0.040 | 0.270 | 443 |
| 10 | Lipoprotein lipases | 84 | 1.30 | 1.06 | -0.033 | 0.261 | 423 |
| 18 | unclassified | 4 | 1.42 | 1.06 | -0.040 | 0.279 | 521 |
| 15 | *Candida rugosa* lipases | 9 | 1.44 | 1.19 | -0.049 | 0.299 | 555 |
| 11 | Cutinases | 20 | 1.45 | 0.96 | -0.044 | 0.290 | 219 |
| 26 | Sfam based on GI 729943  (extracellular lipases and phospholipases) | 7 | 2.14 | 1.21 | -0.012 | 0.290 | 684 |
| 14 | *Moraxella* lipases | 6 | 2.39 | 1.93 | -0.017 | 0.298 | 301 |
| 4 | *Bacillus* lipases | 6 | 2.68 | 1.97 | 0.004 | 0.310 | 211 |

## Table C - All proteins of the database grouped by homologous protein families

n is the number of proteins in the group, charge the charge per residue, turn the relative number of turn- forming residues, sID the superfamily identifier (see table B). Homologous families that belong to cytosolic hydrolases are marked in red. Only homologous families with at least eight proteins were included.

| **sID** | **family** | **n** | **CV-CV’**  **average** | **CV-CV' first quartile** | **charge**  **average** | **turn**  **average** | **length**  **average** |
| --- | --- | --- | --- | --- | --- | --- | --- |
| 30 | Hfam based on GI 729450 | 9 | -1.11 | -1.66 | -0.083 | 0.198 | 247 |
| 24 | Hfam based on GI 3023719 | 8 | -0.65 | -1.23 | -0.074 | 0.211 | 217 |
| 20 | soluble epoxide hydrolases (beta6) | 19 | -0.29 | -0.70 | -0.059 | 0.206 | 303 |
| 23 | Hfam based on GI 126520 | 16 | -0.24 | -0.38 | -0.069 | 0.228 | 307 |
| 20 | soluble haloalkane dehalogenases (beta6) | 23 | -0.19 | -0.61 | -0.064 | 0.222 | 303 |
| 22 | Hfam based on GI 2829433 | 45 | -0.15 | -0.54 | -0.060 | 0.216 | 319 |
| 20 | soluble non-heme peroxidases | 23 | -0.09 | -0.64 | -0.063 | 0.227 | 279 |
| 3 | Non-heme peroxidases | 32 | -0.09 | -0.51 | -0.063 | 0.227 | 276 |
| 24 | Hfam based on GI 22988719 | 9 | 0.01 | -0.08 | -0.059 | 0.224 | 222 |
| 20 | soluble plant epoxide hydrolases | 24 | 0.05 | -0.56 | -0.055 | 0.220 | 318 |
| 16 | *Moraxella* lipase 2 | 53 | 0.16 | -0.49 | -0.055 | 0.227 | 365 |
| 20 | soluble meta cleavage compound hydrolases I | 55 | 0.17 | -0.18 | -0.054 | 0.229 | 281 |
| 20 | soluble haloalkane dehalogenases | 11 | 0.23 | -1.03 | -0.052 | 0.226 | 308 |
| 20 | miscellaneous | 8 | 0.42 | -0.78 | -0.049 | 0.233 | 376 |
| 20 | soluble esterases / lipases / peptidases | 49 | 0.44 | -0.13 | -0.044 | 0.227 | 283 |
| 20 | soluble bacterial epoxide hydrolases II | 9 | 0.50 | -0.33 | -0.042 | 0.230 | 398 |
| 1 | *Rhizomucor* lipases | 32 | 0.52 | 0.11 | -0.052 | 0.243 | 315 |
| 12 | *Acinetobacter* esterases | 26 | 0.62 | -0.12 | -0.039 | 0.227 | 317 |
| 3 | *Haemophilus* lipases | 20 | 0.67 | -0.19 | -0.033 | 0.217 | 304 |
| 20 | soluble bacterial epoxide hydrolases I | 26 | 0.71 | 0.14 | -0.028 | 0.215 | 321 |
| 2 | Mammalian carboxylesterases | 76 | 0.73 | 0.39 | -0.049 | 0.251 | 662 |
| 2 | *Bacillus* esterases | 24 | 0.78 | 0.06 | -0.047 | 0.253 | 527 |
| 9 | Lysosomal acid lipases | 24 | 0.81 | 0.26 | -0.039 | 0.243 | 398 |
| 2 | *Caenorhabditis elegans* esterases II | 17 | 0.84 | 0.31 | -0.038 | 0.239 | 620 |
| 20 | soluble meta cleavage compound hydrolases II | 28 | 0.86 | 0.43 | -0.045 | 0.252 | 288 |
| 19 | microsomal epoxide hydrolases | 30 | 0.86 | 0.47 | -0.039 | 0.241 | 426 |
| 2 | Acetylcholinesterases | 63 | 0.87 | 0.58 | -0.048 | 0.259 | 584 |
| 9 | Gastric lipases | 44 | 0.87 | 0.35 | -0.035 | 0.236 | 439 |
| 3 | *Mycoplasma* lipases | 15 | 0.88 | 0.59 | -0.006 | 0.215 | 268 |
| 3 | *Moraxella* lipase 3 | 23 | 0.89 | 0.36 | -0.030 | 0.229 | 304 |
| 2 | *Caenorhabditis elegans* esterases I | 27 | 0.97 | 0.72 | -0.046 | 0.261 | 559 |
| 2 | Alpha esterases | 53 | 0.98 | 0.71 | -0.038 | 0.248 | 503 |
| 2 | *Drosophila* esterases | 20 | 0.99 | 0.62 | -0.044 | 0.259 | 507 |
| 7 | *Pseudomonas* lipases | 14 | 1.02 | 0.68 | -0.069 | 0.310 | 539 |
| 5 | *Staphylococcus* lipases | 22 | 1.04 | 0.65 | -0.043 | 0.260 | 548 |
| 29 | Hfam based on GI 22986634 | 20 | 1.09 | 0.63 | -0.031 | 0.241 | 428 |
| 24 | Hfam based on GI 32417478 | 36 | 1.10 | 1.02 | -0.039 | 0.256 | 230 |
| 10 | Pancreatic lipases | 49 | 1.24 | 0.88 | -0.039 | 0.267 | 418 |
| 10 | Lipoprotein lipases | 29 | 1.27 | 1.25 | -0.029 | 0.251 | 429 |
| 2 | Mammalian bile salt activated lipases | 15 | 1.36 | 0.54 | -0.047 | 0.288 | 633 |
| 15 | Candida rugosa lipases | 9 | 1.44 | 1.19 | -0.049 | 0.299 | 555 |
| 5 | Burkholderia lipases | 19 | 1.52 | 1.01 | -0.037 | 0.280 | 320 |
| 24 | Hfam based on GI 27808550 | 9 | 1.57 | 1.51 | -0.038 | 0.286 | 342 |

## Table D - Solubility prediction of hydrolases from the PDB

| **PDB ID** | **CV-CV'** | **Source** | **Title** |
| --- | --- | --- | --- |
| 1AUO | -0.420 | *Pseudomonas fluorescens* | Carboxylesterase from *Pseudomonas fluorescens* |
| 1B6G | -1.032 | *Xanthobacter autotrophicus* | Haloalkane dehalogenase at ph 5.0 containing chloride |
| 1BN7 | -0.785 | *Rhodococcus sp.* | Haloalkane dehalogenase from a *Rhodococcus* species |
| 1EHY | -1.625 | *Agrobacterium radiobacter* | X-ray structure of the epoxide hydrolase from *Agrobacterium radiobacter ad1* |
| 1FJ2 | 1.017 | *Homo sapiens* | Crystal structure of the human acyl protein thioesterase 1 at 1.5 a resolution |
| 1GGV | 0.036 | *Pseudomonas putida* | Crystal structure of the c123s mutant of dienelactone hydrolase (dlh) bound with the pms moiety of the protease inhibitor, phenylmethylsulfonyl fluoride (pmsf) |
| 1IUP | 0.076 | *Pseudomonas fluorescens* | Meta-cleavage product hydrolase from *Pseudomonas fluorescens ip01* (cumd) s103a mutant complexed with isobutyrates |
| 1J1I | -0.320 | *Janthinobacterium* | Crystal structure of a his-tagged serine hydrolase involved in the carbazole degradation (carc enzyme) |
| 1JJI | -0.562 | *Archaeoglobus fulgidus* | The crystal structure of a hyper-thermophilic carboxylesterase from the archaeon *Archaeoglobus fulgidus* |
| 1JKM | -0.958 | *Bacillus subtilis* | Brefeldin a esterase, a bacterial homologue of human hormone sensitive lipase |
| 1KEZ | -0.817 | *Saccharopolyspora erythraea* | Crystal structure of the macrocycle-forming thioesterase domain of erythromycin polyketide synthase (debs te) |
| 1LNS | -0.530 | *Lactococcus lactis* | Crystal structure analysis of the x-prolyl dipeptidyl aminopeptidase from *Lactococcus lactis* |
| 1MJ5 | -0.604 | *Sphingomonas paucimobilis* | linb (haloalkane dehalogenase) from *Sphingomonas paucimobilis* ut26 at atomic resolution |
| 1MPX | 0.837 | *Xanthomonas citri* | Alpha-amino acid ester hydrolase labeled with selenomethionine |
| 1MTZ | -0.288 | *Thermoplasma acidophilum* | Crystal structure of the tricorn interacting factor f1 |
| 1NX9 | 0.074 | *Acetobacter pasteurianus* | *Acetobacter turbidans* alpha-amino acid ester hydrolase s205a mutant complexed with ampicillin |
| 1ODT | -0.324 | *Bacillus subtilis* | Cephalosporin c deacetylase mutated, in complex with acetate |
| 1QE3 | -0.695 | *Bacillus subtilis* | pnb esterase |
| 1QO7 | 0.112 | *Aspergillus niger* | Structure of *Aspergillus niger* epoxide hydrolase |
| 1QZ3 | -0.448 | *AlicycloBacillus acidocaldarius* | Crystal structure of mutant m211s/r215l of carboxylesterase est2 complexed with hexadecanesulfonate |
| 1VA4 | -0.407 | *Pseudomonas fluorescens* | *Pseudomonas fluorescens* aryl esterase |
| 1F6W | 1.475 | *Homo sapiens* | Structure of the catalytic domain of human bile salt activated lipase |
| 1EVQ | -0.402 | *AlicycloBacillus acidocaldarius* | The crystal structure of the thermophilic carboxylesterase est2 from *Alicyclobacillus acidocaldarius* |
| 1JFR | 2.223 | *Streptomyces exfoliatus* | Crystal structure of the *Streptomyces exfoliatus* lipase at 1.9a resolution: a model for a family of platelet- activating factor acetylhydrolases |
| 1G42 | -0.663 | *Sphingomonas paucimobilis* | Structure of 1,3,4,6-tetrachloro-1,4-cyclohexadiene hydrolase (linb) from *Sphingomonas paucimobilis* complexed with 1,2-dichloropropane |
| 1JU3 | -0.611 | *Rhodococcus sp. mb1* | Bacterial cocaine esterase complex with transition state analog |
| 1JUD | 0.019 | *Pseudomonas. strain: yl* | l-2-haloacid dehalogenase |
| 1C7I | -0.695 | *Bacillus subtilis* | Thermophylic pnb esterase |
| 1JMK | -0.980 | *Bacillus subtilis* | Structural basis for the cyclization of the lipopeptide antibiotic surfactin by the thioesterase domain srfte |
| 1NM2 | -0.507 | *Streptomyces coelicolor* | Malonyl-coa:acp transacylase |
| 1LZK | -0.832 | *Rhodococcus sp* | Bacterial heroin esterase complex with transition state analog dimethylarsenic acid |
| 1HKH | -1.029 | *Aureobacterium* | Unligated gamma lactamase from an *Aureobacterium* specie |
| 1SFR | 1.942 | *Mycobacterium tuberculosis* | Crystal structure of the *Mycobacterium tuberculosis* antigen 85a protein |
| 1TQH | -1.336 | *Bacillus stearothermophilus* | Covalent reaction intermediate revealed in crystal structure of the *Geobacillus stearothermophilus* carboxylesterase est30 |
| 1VE6 | 1.058 | *Aeropyrum pernix* | Crystal structure of an acylpeptide hydrolase/esterase from *Aeropyrum pernix k1* |
